# TRAIL Receptor Targeting Agents Potentiate PARP Inhibitor Efficacy in Pancreatic Cancer Independently of *BRCA2* Mutation Status

**DOI:** 10.3390/cancers14215240

**Published:** 2022-10-26

**Authors:** Najib Ben Khaled, Katharina Hammer, Liangtao Ye, Ahmed Alnatsha, Sebastian A. Widholz, Ignazio Piseddu, Simon Sirtl, Julia Schneider, Stefan Munker, Ujjwal Mukund Mahajan, Juan José Montero, Joscha Griger, Julia Mayerle, Florian P. Reiter, Enrico N. De Toni

**Affiliations:** 1 Department of Medicine II, University Hospital, LMU Munich, 81377 Munich, Germany; 2 German Cancer Consortium (DKTK), Partner Site Munich, 80336 Munich, Germany; 3 Institute of Molecular Oncology and Functional Genomics, School of Medicine, Technische Universität München, 81675 Munich, Germany; 4 Bavarian Cancer Research Center (BZKF), 91054 Erlangen, Germany; 5 Department of Hepatology, Department of Medicine II, University Hospital Würzburg, 97080 Würzburg, Germany

**Keywords:** apoptosis, *BRCA2*, DNA damage, pancreatic neoplasms, poly(ADP-ribose) polymerase inhibitors, TNF-related apoptosis-inducing ligand

## Abstract

**Simple Summary:**

Research into pancreatic cancer has identified frequent changes in *BRCA* genes, especially in *BRCA2*, occurring in about 5% of patients. *BRCA* proteins help repair damaged DNA. Pancreatic cancers with alterations in *BRCA* genes are sensitive to treatment with PARP inhibitors. The PARP inhibitor olaparib can be used to treat pancreatic cancer with mutations in *BRCA* genes after response to standard platinum-based chemotherapy. Unfortunately, only a few patients with pancreatic cancer have mutations in *BRCA* genes. In this study, we show that the combination of olaparib and TRAIL can be more effective than olaparib alone in killing pancreatic cancer cells. Furthermore, we demonstrate that the combination of olaparib and TRAIL also kills cancer cells without *BRCA2* mutations. Our results suggest a potential new combination therapy of olaparib and TRAIL for pancreatic cancer independent of *BRCA2* mutations and may extend the limited applicability of PARP inhibitors in this disease.

**Abstract:**

Chemotherapy, the standard treatment for pancreatic ductal adenocarcinoma (PDAC), has only a modest effect on the outcome of patients with late-stage disease. Investigations of the genetic features of PDAC have demonstrated a frequent occurrence of mutations in genes involved in homologous recombination (HR), especially in the breast cancer susceptibility gene 2 (*BRCA2*). Olaparib, a poly(ADP-ribose) polymerase (PARP) inhibitor, is approved as a maintenance treatment for patients with advanced PDAC with germline *BRCA1/2* mutations following a platinum-containing first-line regimen. Limitations to the use of PARP inhibitors are represented by the relatively small proportion of patients with mutations in *BRCA1/2* genes and the modest capability of these substances of inducing objective response. We have previously shown that pancreatic cancer with *BRCA2* mutations exhibits a remarkably enhanced sensitivity towards tumor-necrosis-factor-related apoptosis-inducing ligand (TRAIL) receptor-stimulating agents. We thus aimed to investigate the effect of combined treatment with PARP inhibitors and TRAIL receptor-stimulating agents in pancreatic cancer and its dependency on the *BRCA2* gene status. The respective effects of TRAIL-targeting agents and the PARP inhibitor olaparib or of their combination were assessed in pancreatic cancer cell lines and patient-derived organoids. In addition, *BRCA2*-knockout and -complementation models were investigated. The effects of these agents on apoptosis, DNA damage, cell cycle, and receptor surface expression were assessed by immunofluorescence, Western blot, and flow cytometry. PARP inhibition and TRAIL synergized to cause cell death in pancreatic cancer cell lines and PDAC organoids. This effect proved independent of *BRCA2* gene status in three independent models. Olaparib and TRAIL in combination caused a detectable increase in DNA damage and a concentration-dependent cell cycle arrest in the G2/M and S cell cycle phases. Olaparib also significantly increased the proportion of membrane-bound death receptor 5. Our results provide a preclinical rationale for the combination of PARP inhibitors and TRAIL receptor agonists for the treatment of pancreatic cancer and suggest that the use of PARP inhibitors could be extended to patients without *BRCA2* mutations if used in combination with TRAIL agonists.

## 1. Introduction

Pancreatic ductal adenocarcinoma (PDAC) is one of the world’s leading causes of cancer-related death [1,2]. Eighty-five percnt of patients with PDAC are diagnosed with cancer at an advanced stage and do not qualify for curative surgery [3]. Cytotoxic chemotherapy is the mainstay of treatment in patients with advanced PDAC [4]. Unfortunately, both the efficacy and the tolerability of conventional chemotherapy-based treatment regimens are modest, and the prognosis of patients with advanced disease is very poor, with a 5-year survival of <5% [5].

Global efforts to understand the biology of pancreatic cancer have highlighted the role of mutations in genes involved in homologous recombination (HR) DNA repair [6,7]. Among these mutations, alterations in the breast cancer susceptibility gene 2 (*BRCA2*) are among the most frequent and can be identified in approximately 5% of patients [8], although the prevalence can be higher in certain ethnic groups [9,10,11]. HR-deficient (HRD) cancers display an increased sensitivity towards treatment with poly(adenosine diphosphate [ADP]-ribose) polymerase (PARP) inhibitors [12]. PARP inhibitors prevent DNA damage response through blockage of single-strand DNA repair and trapping of the PARP enzymes at sites of DNA lesions. This leads to the replication fork collapse and cell death [12,13,14]. The inability to compensate the PARP inhibitor-induced DNA damage in HRD tumors is responsible for the peculiar sensitivity of this subset of cancers to the action of PARP inhibitors [12].

Olaparib, the first-in-class PARP inhibitor, was investigated as maintenance therapy in patients with advanced PDAC harboring germline *BRCA1* or *BRCA2* alterations, who did not progress after first-line platinum-based chemotherapy [15]. Olaparib improved the primary endpoint of progression-free survival (PFS) as compared to placebo (whose respective median PFSs were 7.4 months and 3.8 months (HR 0.53) [15]) and received regulatory approval [16,17]. However, a survival advantage could not be demonstrated in the setting of this trial [18] and objective responses were modest (overall response rate 23% vs. 12%). Despite its clinical benefit, olaparib is thus regarded as a maintenance therapy able to stabilize the course of pancreatic cancer, rather than active cytotoxic treatment. This, and the relatively small proportion of patients benefitting from its action are limitations to its use in the treatment of pancreatic cancer.

However, there are several preclinical studies indicating that the anticancer potential of PAPRi might be fully exploited as a combination treatment and that their use could be extended to patients not harboring *BRCA2* mutations, thus broadening both their target population and their effectiveness [19]. I this regard, a recent large combinatorial drug screening study in pancreatic, colon, and breast cancer indicates that the anticancer potential of PARP inhibitors could be enhanced by the combination with drugs capable of impinging on cell death mechanisms [20]. Furthermore, PARP1 was shown to reduce the transcription of propoptotic genes to counteract programmed cell death [21]. As a potent inhibitor of PARP1 [22], olaparib and its combination with agents targeting apoptosis signaling could represent an effective therapeutic option in pancreatic cancer.

We have previously shown that pancreatic and colon cancer harboring *BRCA2* alterations exhibit an extremely high sensitivity towards TNF-related apoptosis-inducing ligand (TRAIL)-targeting agents, thereby defining a previously unknown property of *BRCA2* as a regulator of the sensitivity towards cell-death receptor-mediated apotosis [11]. Due to the particular sensitivity of *BRCA2*-deficient cells to both olaparib and TRAIL, and the recent evidence on the influence of PARP-inhibition on apoptosis, we aimed at assessing the effect of combined treatment with olaparib and TRAIL in pancreatic cancer.

## 2. Materials and Methods

### 2.1. Cell Lines and Reagents

Soluble recombinant human TRAIL/TNFS10 was purchased from R&D Systems (Minneapolis, MN, USA). Olaparib and Mitomycin C were obtained from Selleckchem (Houston, TX, USA). Dulanermin was kindly provided by Genentech (San Francisco, CA, USA).

Human pancreatic adenocarcinoma cell lines CAPAN1, CAPAN2, and PATU-S and colorectal carcinoma cell lines DLD1, HCT116, and SW620 were obtained from the Leibniz Institute DSMZ-German Collection of Microorganisms and Cell Cultures (Braunschweig, Germany) and the ATCC (Manassas, VA, USA). The *BRCA2* complemented CAPAN1 clone BRCA2/CIN was kindly provided by Mien-Chie Hung (M.D. Anderson Cancer Center, Houston, TX, USA) [23]. DLD1 BRCA2^KO^ cells were generated by Thomas Hucl and Eike Gallmeier in the laboratory of Scott Kern (Johns Hopkins University, Baltimore, MD, USA) [24]. *BRCA2* gene knockout and complementation were tested upon arrival of cells on the genetic level via sequencing, on the protein level using Western blot and on the functional level by RAD51 focus formation, as described before [25]. All cell lines were tested for mycoplasma and authenticated by the Leibniz Institute DSMZ-German Collection of Microorganisms and Cell Cultures (Braunschweig, Germany) using DNA fingerprinting. PATU-S, DLD1, DLD1 BRCA2^KO^, HCT116 and SW620 cell lines were grown in Dulbecco’s modified Eagle’s medium (DMEM); CAPAN1, CAPAN1 BRCA2/CIN and CAPAN2 in Roswell Park Memorial Institute 1640 medium (RPMI) supplemented with 10% fetal bovine serum and 1% penicillin/streptomycin according to supplier’s conditions at 37 °C with 5% CO_2_. Cells were passaged at a confluency of 80–90% with 0.05% trypsin (*v/v*) in Dulbecco’s Phosphate- Buffered Saline (PBS).

### 2.2. Patient-Derived Organoid Lines

Patient-derived organoids of pancreatic ductal adenocarcinoma (PDAC) were obtained from endoscopic biopsies [26]. Patients had confirmed PDAC diagnosis based on histopathological findings. All patients had to provide written informed consent before inclusion. The local ethics review board approved the study protocol (DRKS00021088). Tumor biopsies were cut into 1–4 mm^3^ pieces and digested with 2 mg/mL collagenase IV, 10 μg/mL DNase I, and 10 μM Rho kinase inhibitor in a medium containing advanced DMEM/F-12 supplemented with 1% HEPES, 1% glutamax, and 0.2% primocin at 37 °C for a maximum of 1 h at constant shaking. The digested tissue was strained over a 100 μm filter. After centrifugation and washing with PBS, the remaining pellet was embedded in Matrigel. Organoids were cultured in medium containing the following factors: B27, 1.25 mM N-acetyl-L-cysteine, 50% (*v/v*) Wnt3a-conditioned medium, 10% (*v/v*) RSPO1-conditioned medium, 0.1 μg/mL recombinant Noggin protein (Peprotech, Hamburg, Germany), 50 ng/mL epidermal growth factor (Peprotech), 10 nM gastrin (Sigma-Aldrich, St. Louis, MO, USA), 100 ng/mL fibroblast growth factor 10 (Peprotech), 10 mM nicotinamide (Sigma-Aldrich), 1 μM prostaglandin E2 (Tocris BioTechne GmbH, Wiesbaden-Nordenstadt, Germany) and 0.5 μM A83-01 (Tocris BioTechne GmbH) based on advanced DMEM/F-12 supplemented with 1% HEPES, 1% glutamax and 0.2% primocin. For passaging, Matrigel domes were dissolved using Cell Recovery Solution (BD Biosciences, Heidelberg, Germany).

### 2.3. Cell Proliferation Assay

A total of 1500 to 2000 cells per well were seeded in 96 multi-well plates and allowed to settle for 24 h. Subsequently, cells were incubated in the presence of various concentrations of the indicated drugs diluted in DMSO. DMSO served as a normalization control. To allow for combination analysis using the Chou–Talalay method [6], cell lines were treated either with (1) control, (2) olaparib, (3) TRAIL or dulanermin, (4) a combination of olaparib and TRAIL or dulanermin. The drug concentrations were determined based on previous experiments or publicly available data from GDSC atlas (https://www.cancerrxgene.org (last accessed on 2 August 2022)) to determine the respective IC50 of each drug alone [27]. Cells were then treated with increasing concentrations reflecting multiples of the IC50 (e.g., ½ × IC50, 1 × IC50, 2 × IC50, etc.). A constant ratio of the combination partners was maintained (e.g., ½ × IC50 of olaparib with ½ × IC50 of TRAIL/dulanermin). After 6 days, cells were washed with PBS and subjected to osmotic lysis in 100 µL of ddH_2_O for 45 min at 37 °C. Subsequently, 0.2% of SYBR green was added to each well, and the optical density was detected on a plate reader (Cytofluor Series 4000, Applied Biosystems, Darmstadt, Germany). Cell proliferation index was calculated as the relative percent change as compared to untreated wells. Data are shown as mean ± standard deviation of three independent experiments. The isobologram and the Chou–Talalay combination index (CI), an established index reflecting the interaction between two drugs, were computed for varying levels of growth inhibition using the CompuSyn software (CompuSyn software, Biosoft) [28]. The CI value determines the lever of drug interaction with a CI < 1 being synergistic, CI = 1 additive and CI > 1 antagonistic, respectively.

### 2.4. Clonogenic Assay

Colonogenic assay was performed as described before [29]. Briefly, 1000 cells were seeded in each well of a 24-well plate. After overnight incubation, cells were exposed to control (DMSO), TRAIL, Olaparib, or their combination in the indicated concentrations. Plates were maintained at 37  °C for 14 days, followed by fixation and staining with 0.2% crystal violet for 30 min (Sigma, St. Louis, MO, USA).

### 2.5. Organoid CellTiter-Glo 3D Viability Assay

To assess organoid viability, Matrigel was dissolved using Cell Recovery Solution and the organoids were dissociated into single cells in TrypLE (Gibco, Thermo Fisher Scientific, Life Technologies Corporation, New York, NY, USA) at 37 °C for 7 min. Subsequently, 2000 cells were plated in 20 μL Matrigel and 80 μL of culture medium and allowed to settle for 24 h. Subsequently, 100 μL culture medium supplemented with 2× concentration of the indicated drugs was added. Six days after treatment, 100 μL CellTiter-Glo^®^ 3D Cell Viability Assay reagent (Promega GmbH, Walldorf, Germany) was added to 100 μL medium in each well and mixed by vigorous pipetting. Plates were incubated at room temperature for 30 min under light protected conditions at constant shaking. Absorbance was measured with a plate reader (BMG Labtech, Ortenberg, Germany). Organoid viability was calculated as the relative percent change as compared to untreated wells. All samples were measured in triplicates and represented as mean ± standard deviation.

### 2.6. Western Blot

Cancer cells were seeded on culture plates and treated as indicated in each experiment. Cells were harvested and lysed on ice using RIPA lysis buffer (Thermo Fisher Scientific) with Pierce Protease Inhibitor Mix (Thermo Fisher Scientific). After centrifugation protein content was determined using Bradford assay. Equal amounts of protein lysate were loaded on 10% SDS-PAGE gels, separated for 15 min at 80 V and for 60 min at 140 V. After gel electrophoresis, proteins were transferred onto PVDF membranes and blocked with Tris-buffered saline with Tween-20 (TBST) containing 5% skim milk (*w/v*) or 5% bovine serum albumin (BSA). Overnight incubation was performed at 4  °C with the following primary antibodies diluted in blocking buffer: CHK1 (#2360), CHK2 (#2662), pCHK1 (#2348), pCHK2 (#2661), Caspase-3 (#9662), Caspase-8 (#9508), Bcl-xL (#2764) from Cell Signaling (Frankfurt am Main, Germany); β-Actin (#A5441) from Sigma Aldrich (Munich, Germany); Bcl-2 (#610539) and Bim (#559685) from BD Biosciences (Heidelberg, Germany); Mcl-1 (#sc-12756), Bad (#sc-8044), Bak (#sc-7873) from Santa Cruz Biotechnology (Heidelberg, Germany) and Bax (#ab7977) from Abcam (Cambridge, UK). The next day, the membrane was probed with HRP-conjugated anti-rabbit or -mouse immunoglobulin G secondary antibodies (GE Healthcare UK Limited) at concentrations of 1:10,000 and incubated for 60 min at room temperature. The band were developed by SuperSignal West Pico Chemiluminescent Substrate (Thermo Scientific, Schwerte, Germany) and imaged with an image acquisition system (ECL ChemoCam Imager, Intas GmbH, Göttingen, Germany).

### 2.7. Flow Cytometry of Surface Receptors

To analyze the TRAIL cell surface receptor expression, cells were cultured on cell culture plates in the presence or absence of olaparib in the indicated concentration. After treatment, cells were washed, detached using trypsin and incubated with or without the respective monoclonal FITC-coupled antibodies: DR4/TRAIL-R1 (ALX-804-297F-T100), DR5/TRAIL-R2 (ALX-804-298F-T100), DcR1/TRAIL-R3 (MAB630), DcR2/TRAIL-R4 (MAB633) from R&D Systems (Minneapolis, MN, USA) and control IgG1 (BD Bioscience, Heidelberg, Germany). Cells immunolabeled with the antibodies were processed using the BD Accuri C6 system (Becton Dickinson, San Jose, CA, USA). The results were analyzed by FlowJo software.

### 2.8. Cell Cycle and Apoptosis Assays

For apoptosis and cell cycle analyses, cells were seeded and allowed to settle overnight, followed by treatment with the indicated substances. After incubation, cells were trypsinized, washed with PBS and stained with propidium iodide. Fluorescence-activated cell sorting was performed using BD Accuri C6 system. The percentage of cells in sub-G1, G0/G1, S or G2/M phase were assessed by FCS Express 6 plus software (De novo software, Pasadena, CA, USA).

### 2.9. Immunofluorescence Microscopy

Cells were seeded and allowed to settle for 24 h, followed by treatment with the indicated substances for 48 h. After incubation, cells were fixed in 4% paraformaldehyde for 10 min, treated with 0.1% Triton X-100 (Invitrogen, Karlsruhe, Germany) for 15 min, wash twice, and then incubated for 30 min with a blocking solution containing 5% BSA in PBS. Samples were then incubated with monoclonal antibodies against γ-H2AX or 53BP1 diluted in blocking solution at 1:200 at 4 °C overnight. Samples were washed twice with PBS containing 0.2% Tween-20 (PBST) and then incubated for 1 h with a secondary goat anti-rabbit antibody conjugated with Alexa Fluor 488 (Invitrogen) at 1:200 dilution. After three washing steps with PBST, samples were mounted with Vectashield (Vector Laboratories, Burlingame, CA, USA) plus Hoechst 33342 (Sigma-Aldrich). Pictures were taken on a Leica fluorescence microscope (Leica Microsystems, Wetzlar, Germany).

### 2.10. Statistics

All analyses were performed using GraphPad Prism 8 Software (GraphPad Software, San Diego, CA, USA). Data are expressed as mean ± standard deviation of at least three independent experiments unless otherwise stated. *p* < 0.05 was considered statistically significant. CI was analyzed as described above [28].

## 3. Results

### 3.1. PARP Inhibition and TRAIL Synergize to Cause Loss of Cell Viability in Pancreatic Cancer Cell Lines

**Figure 1:**
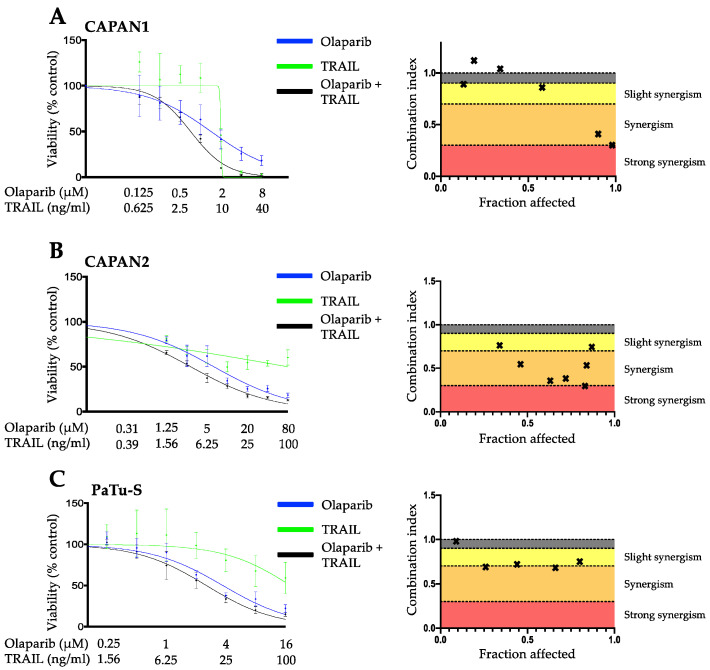
Olaparib and TRAIL synergize in killing pancreatic cancer cell lines. A-C proliferation assays of the effects of olaparib, TRAIL, or their combination on CAPAN1 (**A**), CAPAN2 (**B**), and PaTu-S (**C**). Cells were treated for 6 days with the indicated agents and subsequently analyzed via SYBR green proliferation assay. All experiments were performed in triplicate with error bars representing SEM from three independent experiments. Drug interactions were analyzed using the Chou–Talalay method with a combination index (CI) of <1, 1, and >1 indicating synergistic, additive, and antagonistic drug effects, respectively. In the combination index graphs, dots depict the CI at the respective fraction of cancer cells affected (x-axis, 0.0 = no cells dead, 1.0 = all cells dead).

To assess the hypothesis that the PARP inhibitor olaparib and TRAIL might exert a synergistic anticancer effect in pancreatic cancer cells, we selected three well-characterized PDAC cell lines with different driver mutation patterns: CAPAN1 (*KRAS*, *CDKN2A*, *SMAD4*, *TP53*), CAPAN2 (*KRAS*) [30] and PATU-S (*KRAS*, *TP53*, *SMAD4*) [31]. Cancer cell lines were treated with control, olaparib, TRAIL, and olaparib plus TRAIL in increasing concentrations, representing multiples of the IC50 of each drug at a constant ratio (e.g., ½ × IC50 olaparib: ½ × IC50 TRAIL, 1 × IC50 olaparib: 1 × IC50 TRAIL, etc.) to determine the cell proliferation index. Combination index (CI) blots were generated using the Chou-Talay model [28]. Drug interactions with a CI < 1, 1, and > 1 indicated synergistic, additive, and antagonistic drug effects, respectively. Olaparib and TRAIL exhibited a potent synergistic antineoplastic activity with CI values below 0.7 (Figure 1) in CAPAN1, CAPAN2, and PaTu-S pancreatic cancer lines. This synergism was observed at clinically viable plasma concentrations and was maintained at a high fraction of cells affected, which is crucial for oncological pharmacotherapy [28]. To confirm these findings, we performed clonogenic assays in the cell line CAPAN1. The results from the viability assay were confirmed by a dose-dependent reduction in the number and size of colonies in CAPAN1 upon incubation with olaparib and TRAIL when compared to olaparib or TRAIL alone (Supplementary Figure S1).

### 3.2. The Synergistic Interaction between Olaparib and TRAIL Is Independent of BRCA2 Mutational Status

**Figure 2:**
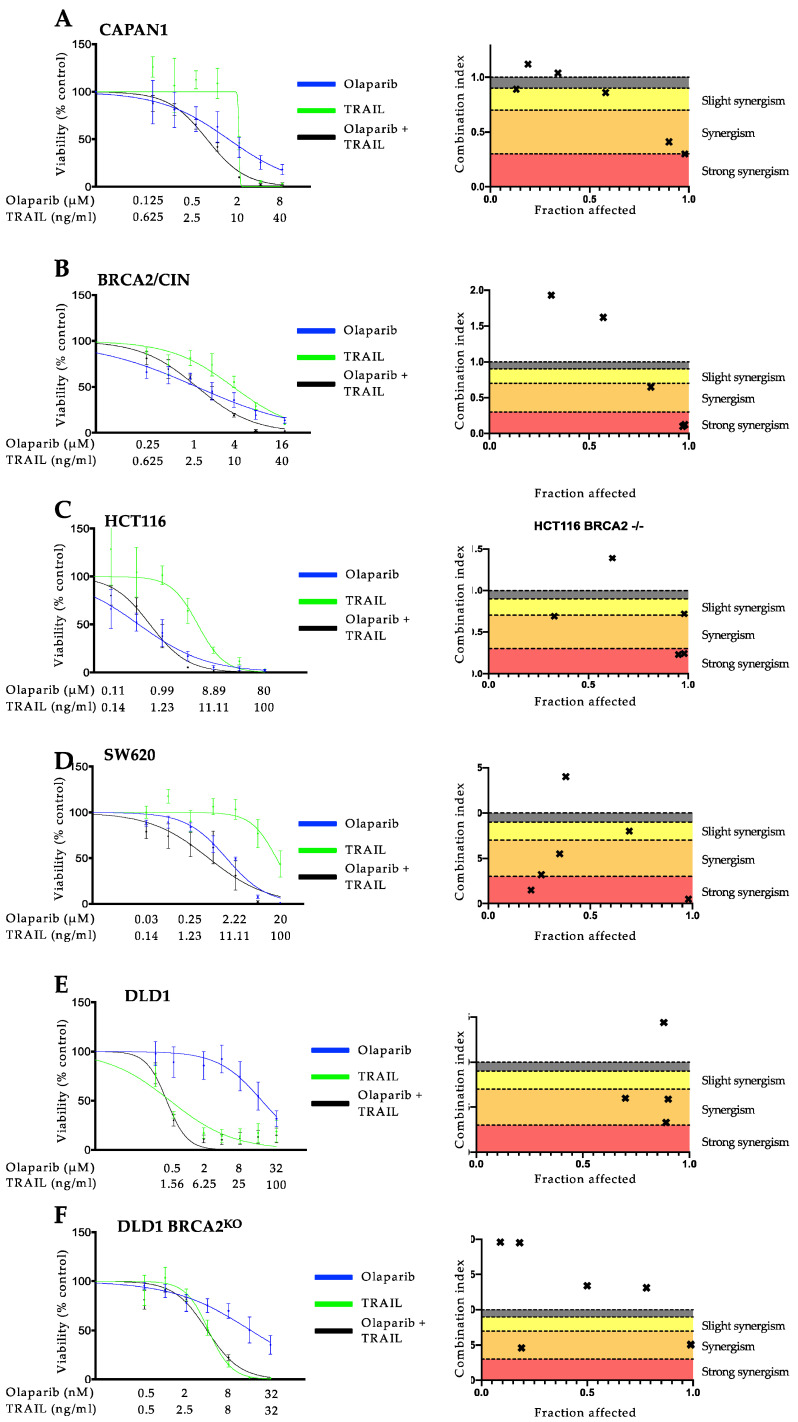
Olaparib and TRAIL synergize in cancer cells independently of *BRCA2* status. A-B Proliferation assays of the effects of olaparib, TRAIL, or their combination on BRCA2 deficient CAPAN1 (**A**), and the respective syngenic BRCA2/CIN cell line (**B**) complemented to express wild-type BRCA2. C-D Proliferation assays of the effects of olaparib, TRAIL or their combination on *BRCA2* deficient HCT116 (**C**), and *BRCA2* proficient SW620 (**D**). E-F Proliferation assays of the effects of olaparib, TRAIL or their combination on wild-type DLD1 cells (**E**) vs. the homozygous *BRCA2* knockout colorectal cancer cell line DLD1 termed DLD1 BRCA2^KO^ (**F**). Cells were treated for 6 days with the indicated agents and subsequently analyzed via SYBR green proliferation assay. All experiments were performed in triplicate with error bars representing SEM from three independent experiments. Drug interactions were analyzed using the Chou–Talalay method with a combination index (CI) of <1, 1, and >1 indicating synergistic, additive, and antagonistic drug effects, respectively. In the combination index graphs, dots depict the CI at the respective fraction of cancer cells affected (x-axis, 0.0 = no cells dead, 1.0 = all cells dead).

**Figure 3:**
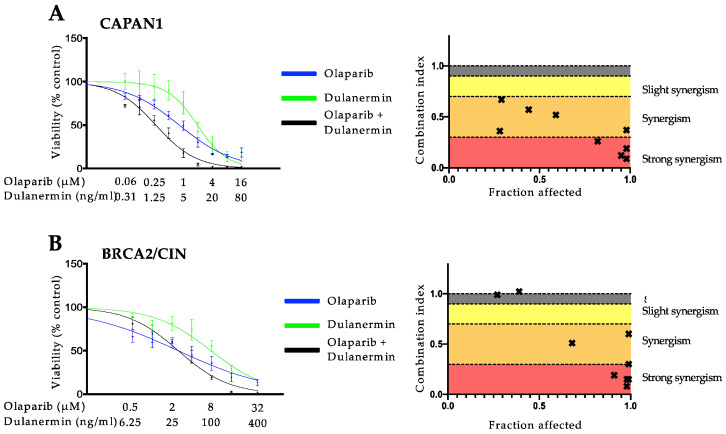
Combined olaparib and the clinically tested TRAIL targeting compound dulanermin synergize in pancreatic cancer cells independently of *BRCA2* status. Proliferation assays of the effects of olaparib, dulanermin, or their combination on *BRCA2* deficient CAPAN1 (**A**) and BRCA2/CIN (**B**). Cells were treated for 6 days with the indicated agents and subsequently analyzed via SYBR green proliferation assay. All experiments were performed in triplicate with error bars representing SEM from three independent experiments. Drug interactions were analyzed using the Chou–Talalay method with a combination index (CI) of <1, 1, and >1 indicating synergistic, additive, and antagonistic drug effects, respectively. In the combination index graphs, dots depict the CI at the respective fraction of cancer cells affected (x-axis, 0.0 = no cells dead, 1.0 = all cells dead).

Since *BRCA2* mutations are well known to increase the sensitivity toward PARP inhibition and, as we previously showed, to TRAIL-receptor-targeting agents [12,13,25], we next aimed at assessing the effect of *BRCA2* gene status on the interaction of these agents and whether this synergy is specific to pancreatic cancer. To this end, we used three different models, consisting of pairs of *BRCA2*-deficient or proficient cells: (1) CAPAN1, which lacks a functional *BRCA2* allele [32,33], and the respective syngenic BRCA2/CIN cell line complemented to express wild-type *BRCA2* [23]; (2) the homozygous *BRCA2* knockout colorectal cancer cell line DLD1 termed DLD1 BRCA2^KO^ vs. wild-type DLD1 cells [24] and (3) *BRCA2* deficient HCT116 vs. *BRCA2-*proficient SW620 [30]. First, we examined the antineoplastic activity of olaparib, TRAIL, and the DNA cross-linking agent mitomycin C, which was used as a control [34,35,36]. As expected, and in line with previous studies [12,25,34], cells with *BRCA2* deficiency showed a higher sensitivity towards olaparib, TRAIL (Supplementary Figure S2A), and mitomycin C used individually (Supplementary Figure S3A) as compared to the *BRCA2* proficient cells with IC50 ratios ranging from 0.07–0.68 for olaparib and TRAIL (Supplementary Figure S2B) and 0.06 to 0.55 for mitomycin C (Supplementary Figure S3B). Unexpectedly, however, when used in combination, olaparib and TRAIL showed considerable synergistic activity in all the cell lines tested independently of the *BRCA2* gene status in cell lines from both tumor entities (Figure 2). The strongest synergistic effect could be seen in the *BRCA2* proficient pancreatic cancer cell line CAPAN1 BRCA2/CIN with CI values of 0.1 at a high fraction of cells affected (Figure 2B). These results were validated by colony formation assay (Supplementary Figure S1). Dulanermin is another soluble recombinant human TRAIL ligand binding to death receptor (DR) 4 and DR5 [37]. Dulanermin has been studied in many clinical trials as monotherapy or in combination with chemotherapy and has proven to be safe in humans [38,39,40,41,42,43,44,45,46]. To investigate whether the observed synergism also occurs with olaparib and dulanermin, two agents that have been extensively tested in clinical trials, CAPAN1 and BRCA2/CIN cells, were incubated with olaparib, dulanermin, or their combination. Notably, the use of dulanermin as a combination partner to olaparib also showed potent synergism in the cells tested, with CI values ranging from 0.1 to 0.7 (Figure 3). Due to the specific potential clinical relevance of the use of olaparib-based combinations in pancreatic cancer and the fact that olaparib and TRAIL demonstrated the strongest synergistic effect in CAPAN1 and BRCA2/CIN, these cells were used for further mechanistic studies.

### 3.3. Combined Olaparib and TRAIL Enhance S/G2 Phase Cell Cycle Arrest and Apoptosis

**Figure 4:**
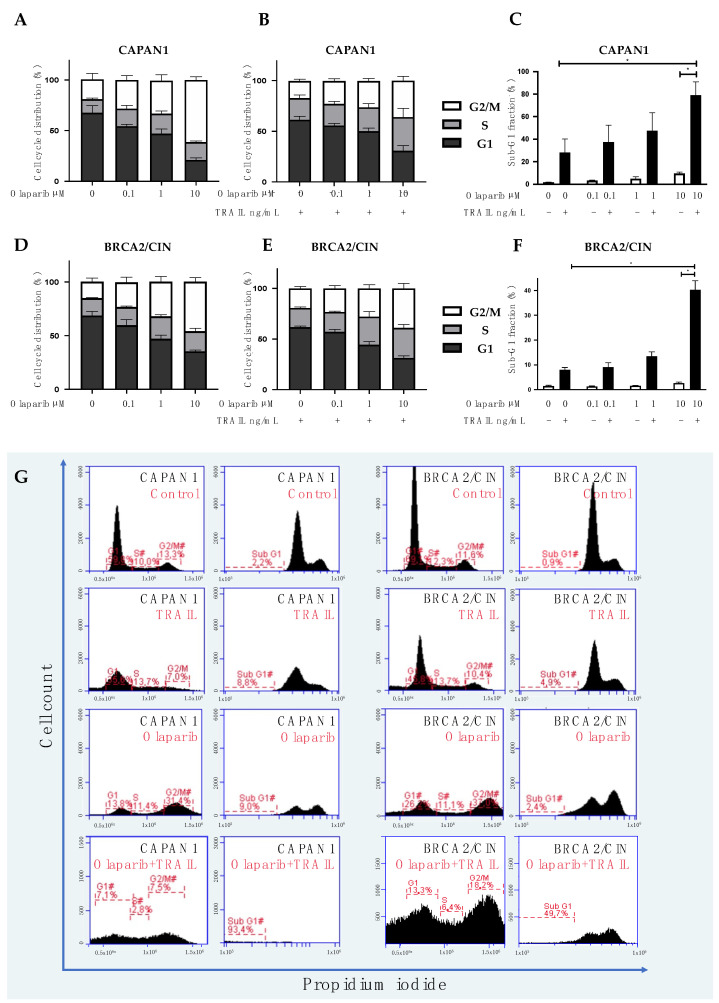
Combined olaparib and TRAIL enhance S/G2 phase cell cycle arrest and apoptosis. CAPAN1 (**A**–**C**), and the wild-type BRCA2 expressing BRCA2/CIN treated with various doses of olaparib alone (**A**,**D**) or in combination with TRAIL (10 ng/mL) (**B**,**C**,**E**,**F**) for 48 h. Figure 4A,B,D,E depict the percentage of cells in the respective cell cycle phase from all viable cells, i.e., non-sub-G1 cells. Figure 4C,F depict the percentage of cells in sub-G1 from all cells. Figure (**G**): Representative flow cytometry histograms of CAPAN1 and BRCA2/CIN cells after incubation with control, olaparib (10 µM), TRAIL (10 mg/mL), or its combination. Error bars represent mean  ±  SEM from at least three experiments. * = statistical significance, *p* < 0.05.

To elucidate the mechanisms underlying the synergistic interaction to kill cancer cells, we separately assessed the effect of combined substances on the cell cycle and cell death. CAPAN1 and CAPAN1 BRCA2/CIN were incubated for 48 h with olaparib or TRAIL and subjected to PI staining and flow cytometry. Gates were set to calculate cells in cell cycle phases sub-G1, G1, S, and G2/M. As expected, increasing concentrations of olaparib caused a corresponding increase in the proportion of cells in the G2/M-cell cycle phase (Figure 4A), whereas TRAIL did not affect cell cycle (Supplementary Figure S4). Interestingly, however, when combined, olaparib and TRAIL caused a profound and concentration-dependent increase in the cell population in both the G2/M- and S phases of the cell cycle, indicating an interaction leading to cell cycle arrest (Figure 4B). In addition, when combined, olaparib and TRAIL led to a strong and dose-dependent increase in apoptosis, as shown by the assessment of the fraction of sub-G1 events (Figure 4C). The effect was more pronounced in CAPAN1 (Figure 4A–C), probably reflecting the genomic vulnerability of this *BRCA2*-deficient cell line.

**Figure 5:**
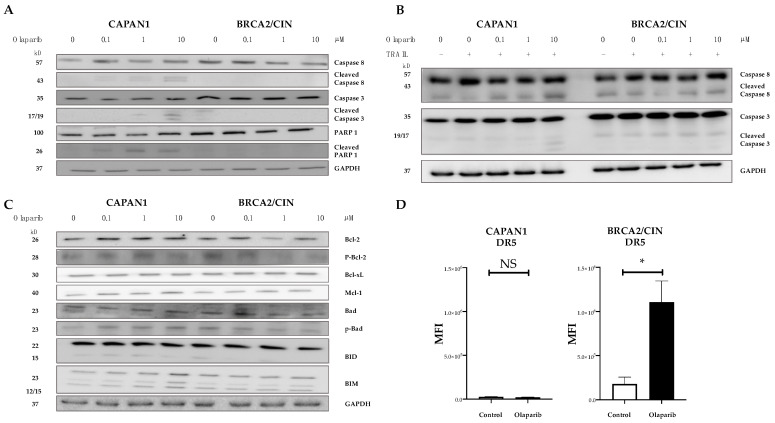
Olaparib influences death receptor expression and caspase activation. (**A**–**C**): Western blot analysis to assess the expression levels of the indicated regulators of apoptosis in BRCA2 deficient CAPAN1 vs. wild-type BRCA2 expressing BRCA2/CIN treated with various doses of olaparib alone or in combination with TRAIL (10 ng/mL) for 48 h. Data show representative results from three experiments. GAPDH served as a loading control. (**D**): Flow cytometry of TRAIL surface receptor 2 (death receptor 5, DR5) in BRCA2 deficient CAPAN1 vs. wild-type BRCA2 expressing BRCA2/CIN treated with 1 μM olaparib for 6 days. Error bars represent mean  ±  SEM from at least three experiments. * = statistical significance, *p* < 0.05, NS = non-significant, MFI = median fluorescence intensity.

Assessment of the surface expression of TRAIL receptors showed that olaparib increased the staining intensity of membrane-bound death receptor (DR) 5 in the context of *BRCA2* proficiency (Figure 5D) but did not affect membrane staining of DR4 and the two known membrane-bound decoy receptors for TRAIL (DcR1 and DcR2–Supplementary Figure S5) [37,47,48]. This is in line with previous findings showing that PARP inhibition causes the upregulation of DR5 in leukemia and ovarian and lung cancer [47]. Consistently, olaparib caused a concentration-dependent cleavage of caspase-8 (which is activated upon stimulation of receptor-mediated apoptosis) and of caspase-3 in *BRCA2*-deficient CAPAN1 cells (Figure 5A), an effect that was further increased by adding TRAIL to olaparib (Figure 5B). However, since the highest apoptotic fraction could be observed in the context of *BRCA2* deficiency (Figure 4C), this increased activation of caspase-8 could also be mediated through already ongoing apoptosis due to DNA damage accumulating upon treatment with olaparib. Olaparib did not increase the baseline expression of procaspase-8 or procaspase -3 (Figure 5A), nor did it affect the expression or phosphorylation of key proteins of the mitochondrial pathway, such as the proapoptotic regulators Bad, Bid, and Bim or the antiapoptotic Bcl-2, Bcl-xL and Mcl-1 (Figure 5C).

Taken together, these data indicate that stimulation of the death receptor, considered to exert a purely pro-apoptotic function, may cause cell cycle changes if combined with olaparib. In addition, although olaparib did not affect apoptosis alone, it increased TRAIL-mediated recruitment of caspase 8 without affecting the intrinsic apoptotic pathway, an effect that could be attributable to the upregulation of DR5.

### 3.4. Influence of Olaparib and TRAIL on DNA Damage Repair

**Figure 6:**
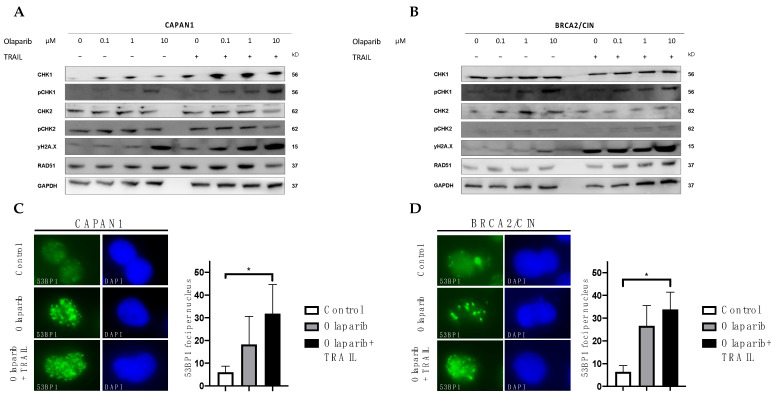
Influence of Olaparib and TRAIL on DNA damage repair. (**A**,**B**): Western blot analysis to assess the expression levels of the indicated DNA damage proteins in *BRCA2*-deficient CAPAN1 vs. wild-type *BRCA2* expressing BRCA2/CIN treated with various doses of olaparib alone or in combination with TRAIL for 48 h. Data show representative results from three experiments. GAPDH served as a loading control. (**C**,**D**): 53BP1 immunostaining of BRCA2 deficient CAPAN1 vs. wild-type BRCA2 expressing BRCA2/CIN treated with 10 μM olaparib +/− 10 ng/mL TRAIL or dimethyl sulfoxide (DMSO) control for 48 h. Quantification of 53BP1 foci per nucleus was based on at least 10 nuclei per sample. Error bars represent mean  ±  SD. * = statistical significance, *p* < 0.05.

Olaparib blocks PARP-dependent DNA single-strand break repair, causing replication-induced DNA damage followed by replication fork collapse [12,13,14]. Furthermore, olaparib inhibits the PARylation of multiple substrates of the PARP enzymes, which is a crucial step in the regulation of DNA repair [12,13,14,48]. To assess whether the synergism between olaparib and TRAIL arises from increased DNA damage, we first examined the levels of phosphorylated H2AX (γH2AX), a sensible marker of DNA double-strand breaks. PARP inhibition with olaparib induced DNA damage with subsequent activation of the DNA damage response, as evidenced by an increase in γH2AX (Figure 6A,B; left panel). As expected, this effect was more pronounced in the *BRCA2*-deficient CAPAN1 (Figure 6A; left panel) as compared to the *BRCA2* proficient BRCA2/CIN cell (Figure 6B; left panel). Interestingly, the combination of olaparib and TRAIL led to a strong increase in DNA damage, which was seen independent lyof the *BRCA2* gene status (Figure 6A,B; right panels). These results could be validated by γH2AX immunostaining, where an increased formation of γH2AX foci upon combined olaparib and TRAIL was evident (Supplementary Figure S6) independent of *BRCA2* status.

To elucidate the molecular mechanisms behind the activated DNA damage response, we aimed to assess the activation and expression of key regulators of the DDR pathway. RAD51 is a key protein orchestrating homologous recombination, whereas 53BP1 has an essential role in the error-prone non-homologous end-joining (NHEJ) repair machinery. Interestingly, olaparib alone or in combination with TRAIL strongly upregulated 53BP1 focus formation (Figure 6C,D), indicating an increase in NHEJ DNA repair. Olaparib alone or combined with TRAIL did not influence the expression of RAD51 (Figure 6A,B). Olaparib alone led to an increased formation of RAD51 foci (Supplementary Figure S7). However, the addition of TRAIL to olaparib did not further increase RAD51 focus formation. In addition to the immunofluorescence experiments, we also performed Western blot analysis of replication protein A (RPA), which is critical in the regulation of homologous recombination [49,50]. We could not detect hyperphosphorylation of RPA upon combined olaparib and TRAIL (Supplementary Figure S8). In the next step, we examined the effect of olaparib alone or combined with TRAIL on replication checkpoint responses. We could observe an increase in DNA damage-induced phosphorylation of CHK1-Ser345 indicating activation of CHK1 in both cell lines (Figure 6A,B). This is in accordance with our previous results since pCHK1 is an important regulator of the cell cycle and mediates the G2/M transition [51].

In summary, olaparib combined with TRAIL leads to an accumulation of DNA damage resulting in activation of the NHEJ repair and phosphorylation of CHK1 with subsequent cell cycle arrest and apoptosis.

### 3.5. Combination of Olaparib and TRAIL Enhances Antitumor Activity in Patient-Derived Pancreatic Cancer Organoids

**Figure 7:**
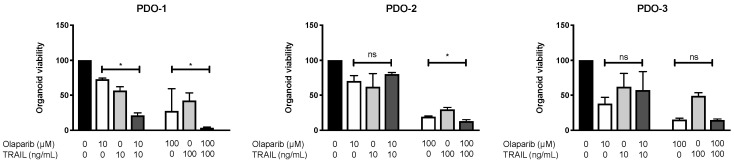
The combination of olaparib and TRAIL enhances antitumor activity in patient-derived pancreatic cancer organoids. CellTiter-Glo assay of organoids derived from three patients with pancreatic cancer (PDO1, PDO2, PDO3) treated with the indicated substances or dimethyl sulfoxide (DMSO) control for 6 days. Error bars represent mean  ±  SEM from three experiments. * = statistical significance, *p* < 0.05, ns = non-significant.

Pancreatic cancer organoids maintain genomic and morphologic characteristics of the tumor of origin and can predict therapeutic responses to drugs [52]. We, therefore, assessed whether the enhanced antitumoral activity of olaparib and TRAIL observed in established cell lines could be validated in patient-derived organoids (PDOs). To this aim, we established three PDOs from endoscopic ultrasound-guided biopsies. The PDOs were exposed to either TRAIL or olaparib or their combination at a low (Olaparib 10 µM, TRAIL 10 ng/mL) and high dose (Olaparib 100 µM, TRAIL 100 ng/mL). Olaparib and TRAIL significantly inhibited the proliferation of two organoids derived from pancreatic cancer patients compared with olaparib alone, (Figure 7, PDO-1 and PDO-2), while in a third organoid (PDO-3) the addition of TRAIL did not improve the efficacy of olaparib (Figure 7, PDO-3).

## 4. Discussion

In the last decade, PARP inhibitors have paved their way into clinical practice with varying degrees of efficacy across different tumor types. In ovarian cancer, PARP inhibitors have revolutionized the treatment of patients with HR-deficient tumors. Even in patients with advanced disease, exceptionally long and durable responses and prolonged survival have been reported along with modest treatment-related toxicity [53,54]. However, PARP inhibitors are not as effective in pancreatic cancer, where they are used as maintenance treatment in patients with germline *BRCA1/2* mutations after first-line platinum-based chemotherapy. Furthermore, their utility is limited by the small proportion of patients harboring mutations in HRD, with *BRCA2* being the most frequently observed mutation in around 5% of patients. We have previously shown that pancreatic cancers with *BRCA2* mutations display notably increased susceptibility towards TRAIL receptor-stimulating agents [25]. This led us to ask whether a combination of PARP inhibitors and TRAIL could be a feasible therapeutic strategy in pancreatic cancer. In our study, the combination of olaparib and TRAIL resulted in potent synergistic antitumor activity in pancreatic cancer cell lines and patient-derived organoids. Notably, the synergism could be observed not only in *BRCA2*-deficient cancer cells but also in their *BRCA2* proficient counterparts in three independent models.

TRAIL agonists initiate the apoptosis cascade through binding to the death receptors DR4 and DR5 [55,56]. The rationale for using TRAIL targeting agents in cancer treatment was the peculiar ability of these compounds to kill tumor cells while leaving healthy tissue unharmed in preclinical models [37,55,56,57]. Early phase clinical trials with TRAIL receptor agonists confirmed the good safety profile of these agents showing signs of efficacy [38,39,40]. However, with one notable exception [46], these agents failed to prove a clinical benefit in large randomized clinical trials in unselected patient cohorts [41,42,43,44,45]. Several factors contributed to the failure of TRAIL therapies in the past. First, one suggested reason for the lack of efficacy of TRAIL agonists is their low-affinity [58,59] as well as suboptimal activation of TRAIL death receptor signaling and apoptosis in vivo [60,61]. Development of more potent drugs is on the way but increasing potency might also increase toxicity. The more potent TRAIL targeting compound TAS266, for example, showed unexpected, severe hepatotoxicity in a phase I clinical trial [62]. Combining low potency, low toxicity TRAIL agonists such as dulanermin with olaparib might represent a way of increasing their respective efficacy without compromising tolerability. Second, there is a lack of effective biomarkers that can predict response to TRAIL agonists. Our discovery that *BRCA2* mutations identify patients susceptible to the action of TRAIL agonists suggests that TRAIL could be used in these patients in analogy to the way the recognition of the relevance of microsatellite instability (MSI) preceded the success of checkpoint inhibitors (CPI) in treating MSI-high colorectal cancer patients after the failure of CPI in unselected cohorts [25,63,64,65]. Third, low cell surface expression of death receptors may also contribute to reduced activity of TRAIL agonists [66]. In previous work, we showed that loss of TRAIL receptors is a common feature in pancreatic cancer [67]. The PARP inhibitor olaparib contributed to enhanced TRAIL-mediated apoptosis by increasing cell surface expression of DR5 and downstream activation of caspases. We therefore confirm the notion that PARP inhibitors prime apoptosis [47]. Besides representing a rationale for combining PARP inhibitors and TRAIL-targeting agents, our findings are relevant to the possibility of combining Olaparib to other novel clinically viable apoptosis-modulating drugs, including Bcl2 inhibitors or nuclear factor erythroid 2-related factor 2 (*NRF2*) inhibitors, for treatment of PDAC. *NRF2* is highly expressed in PDAC [68]; its inhibition was shown to sensitize PDAC cells to apoptosis [69] and synergized with cytotoxic therapy to cause cell death in cancer cells across various entities [70,71,72,73]. A combination of PARP inhibitors and apoptosis-inducing agents such as *NRF2* inhibitors could thus represent a feasible strategy in cancer treatment. In this context, mechanistic evaluations of the effect of apoptosis-inducing agents on DNA damage and repair mechanisms, such as reporter-based quantification of NHEJ and HR pathways [74,75,76,77], may help identify the ideal combination partner for PARP inhibitors in tissue-specific contexts.

The present findings on the synergistic interaction between olaparib and TRAIL have, however, additional relevance. Although the use of PARP inhibitors represents the first instance to exploit synthetic lethality in pancreatic cancer, their use is by nature limited by (1) the relatively low occurrence of mutations in *BRCA* genes [8] and (2) inevitable and frequent drug resistance [78]. Our results suggest that the synergy between olaparib and TRAIL is independent of the *BRCA2* mutational status. Therefore, not only could the combination of these agents greatly improve their individual effects, while possibly leaving the toxicity profile unchanged, but the combination of olaparib and TRAIL could also greatly extend the number of patients who could potentially benefit from this treatment, switching a PARP-inhibitor from a resistant to sensitive phenotype by the addition of TRAIL. A PARP inhibitor-TRAIL combination treatment could therefore represent a feasible strategy to broaden the clinical use of this drug class and counteract inherent or acquired resistance.

## 5. Conclusions

Our results provide a preclinical rationale for the combination of PARP inhibitors and TRAIL receptor agonists independent of *BRCA2* mutation status in pancreatic cancer and may extend the limited applicability of PARP inhibitors in this disease.

## Data Availability

External data sharing requests will be considered for additional topics not already pursued by the investigators. Researchers can submit a request for data with a research proposal and a data-sharing agreement to the corresponding author for review. Data availability will begin one year and end three years after publication.

## Supplementary Materials

The following supporting information can be downloaded at: https://www.mdpi.com/article/10.3390/cancers14215240/s1, Figure S1: clonogenic assays; Figure S2: *BRCA2* deficiency enhances the sensitivity of cancer cells towards PARP inhibition or TRAIL; Figure S3: *BRCA2* deficiency enhances the sensitivity of cancer cells towards mitomycin C; Figure S4: TRAIL treatment cell cycle analysis; Figure S5: flow cytometry of TRAIL surface receptors; Figure S6: γ-H2AX immunostaining; Figure S7: RAD51 immunostaining; Figure S8: western blot analysis to assess the expression levels of pRPA32.
