# Supplementary material for: TRAIL Receptor Targeting Agents Potentiate PARP Inhibitor Efficacy in Pancreatic Cancer Independently of BRCA2 Mutation Status
Source: Cancers (Basel). 2022 Oct 26;14(21):5240. doi: 10.3390/cancers14215240 (PMC9655638; doi:10.3390/cancers14215240)
Supplement: Supplementary file 1 [file cancers-14-05240-s001.zip › Figure S1.pdf]

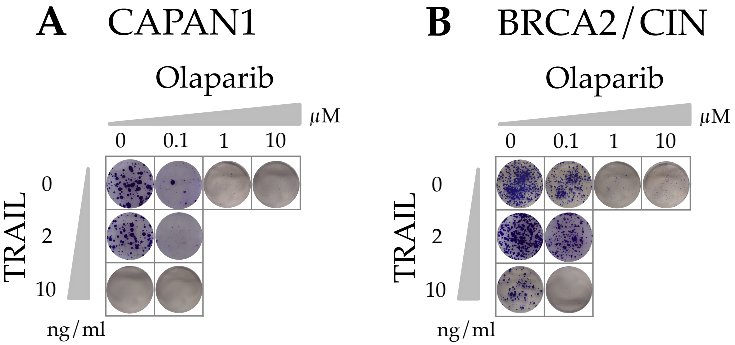

Figure S1. CAPAN1 and the wild-type BRCA2 expressing BRCA2/CIN were treated with various doses of olaparib, TRAIL or its combination. Cells were treated for 14 days with the indicated agents and subsequently subjected to crystal violet staining. All experiments were performed in duplicates.
