# Supplementary material for: TRAIL Receptor Targeting Agents Potentiate PARP Inhibitor Efficacy in Pancreatic Cancer Independently of BRCA2 Mutation Status
Source: Cancers (Basel). 2022 Oct 26;14(21):5240. doi: 10.3390/cancers14215240 (PMC9655638; doi:10.3390/cancers14215240)
Supplement: Supplementary file 1 [file cancers-14-05240-s001.zip › Figure S2.pdf]

A

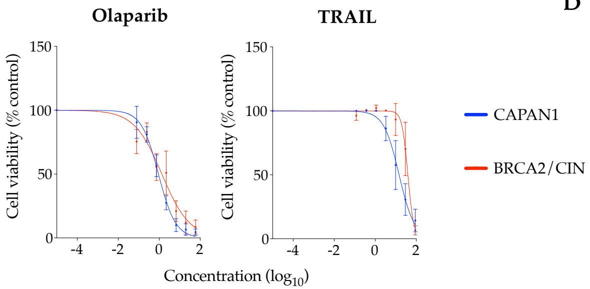

B

Compound (unit)	IC50		IC50 ratio
	CAPAN1	CAPAN1 BRCA2/CIN	
Olaparib (μm)	0.93	1.36	0.68
TRAIL (ng/ml)	14.73	39.51	0.37

C

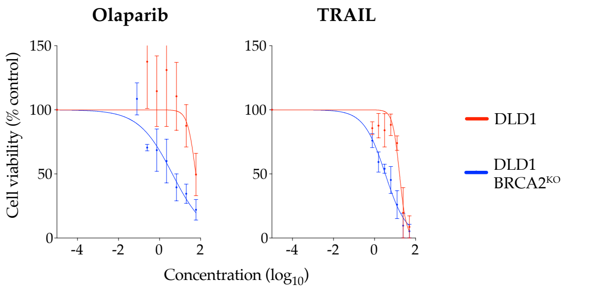

D

Compound (unit)	IC50		IC50 ratio
	DLD1 BRCA2^{KO}	DLD1	
Olaparib (μm)	4.25	58.74	0.07
TRAIL (ng/ml)	3.34	16.53	0.20

E

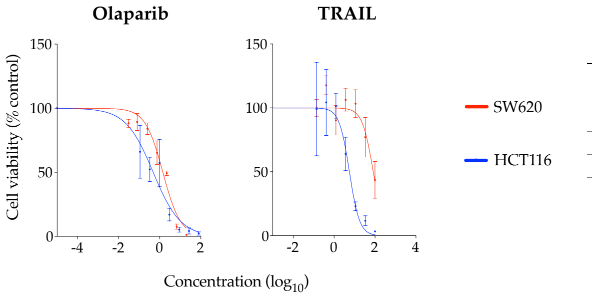

F

Compound (unit)	IC50		IC50 ratio
	HCT116	SW620	
Olaparib (μm)	0.48	1.42	0.34
TRAIL (ng/ml)	5.63	82.46	0.07

Figure S2. BRCA2 deficiency enhances the sensitivity of cancer cells towards PARP inhibition or TRAIL. SYBR green proliferation assay of BRCA2 deficient CAPAN1 and the respective syngenic BRCA2/CIN cell line complemented to express wild-type BRCA2 (A, B), homozygous BRCA2 knockout colorectal cancer cell line DLD1 termed DLD1 BRCA2^{KO} vs. wild-type DLD1 cells (C, D), BRCA2 deficient HCT116 and BRCA2 proficient SW620 (E, F) after 6 days of treatment with olaparib or TRAIL in increasing concentrations. All experiments were performed at least in duplicates with error bars representing SEM from independent experiments. B, D, F: IC50 values obtained for each compound tested and their ratio, defined as IC50 in the context of BRCA2 deficiency divided by IC50 in the context of BRCA2 proficiency.
