# Supplementary material for: TRAIL Receptor Targeting Agents Potentiate PARP Inhibitor Efficacy in Pancreatic Cancer Independently of BRCA2 Mutation Status
Source: Cancers (Basel). 2022 Oct 26;14(21):5240. doi: 10.3390/cancers14215240 (PMC9655638; doi:10.3390/cancers14215240)
Supplement: Supplementary file 1 [file cancers-14-05240-s001.zip › Figure S3.pdf]

A

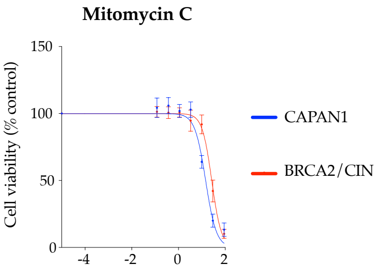

B

Compound (unit)	IC50 CAPAN1	IC50 CAPAN1 BRCA2/CIN	IC50 ratio
Mitomycin C (nm)	14.63	26.77	0.55

C

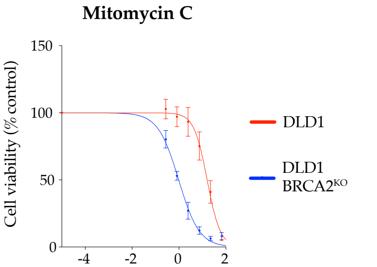

D

Compound (unit)	IC50 DLD1 BRCA2^{KO}	IC50 DLD1	IC50 ratio
Mitomycin C (nm)	0.98	16.04	0.06

Figure S3. BRCA2 deficiency enhances the sensitivity of cancer cells towards mitomycin C. SYBR green proliferation assay of BRCA2 deficient CAPAN1 and the respective syngenic BRCA2/CIN cell line complemented to express wild-type BRCA2 (A, B) and homozygous BRCA2 knockout colorectal cancer cell line DLD1 termed DLD1 BRCA2^{KO} vs. wild-type DLD1 cells (C, D) after 6 days of treatment with olaparib or TRAIL in increasing concentrations. All experiments were performed at least in duplicates with error bars representing SEM from independent experiments. B, D: IC50 values obtained for each compound tested and their ratio, defined as IC50 in the context of BRCA2 deficiency divided by IC50 in the context of BRCA2 proficiency.
