# Supplementary material for: TRAIL Receptor Targeting Agents Potentiate PARP Inhibitor Efficacy in Pancreatic Cancer Independently of BRCA2 Mutation Status
Source: Cancers (Basel). 2022 Oct 26;14(21):5240. doi: 10.3390/cancers14215240 (PMC9655638; doi:10.3390/cancers14215240)
Supplement: Supplementary file 1 [file cancers-14-05240-s001.zip › Figure S4.pdf]

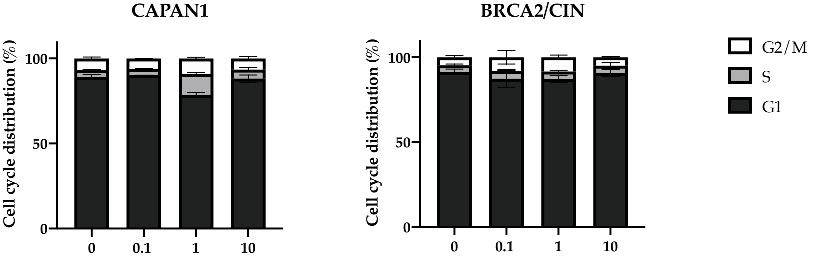

Figure S4. CAPAN1 and the wild-type BRCA2 expressing BRCA2/CIN were treated with various doses of TRAIL for 48 h. Error bars represent mean ± SEM from two experiments.
