# Supplementary material for: TRAIL Receptor Targeting Agents Potentiate PARP Inhibitor Efficacy in Pancreatic Cancer Independently of BRCA2 Mutation Status
Source: Cancers (Basel). 2022 Oct 26;14(21):5240. doi: 10.3390/cancers14215240 (PMC9655638; doi:10.3390/cancers14215240)
Supplement: Supplementary file 1 [file cancers-14-05240-s001.zip › Figure S5.pdf]

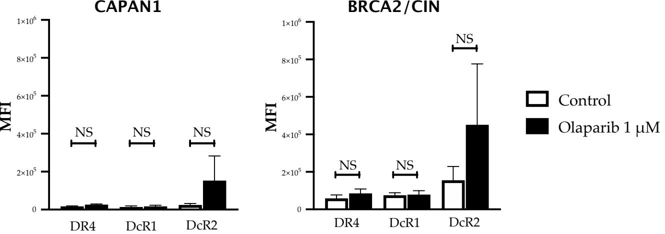

Figure S5. Flow cytometry of TRAIL surface receptors. Death receptor 4 (DR4), membrane-bound decoy receptor 1 (DcR1) and decoy receptor 2 (DcR2) were stained in BRCA2 deficient CAPAN1 vs. wild-type BRCA2 expressing BRCA2/CIN treated with 1 μM olaparib for 6 days. Error bars represent mean ± SEM from at least three experiments. NS = non-significant, P ≥ 0.05, MFI=median fluorescence intensity.
