# Supplementary material for: TRAIL Receptor Targeting Agents Potentiate PARP Inhibitor Efficacy in Pancreatic Cancer Independently of BRCA2 Mutation Status
Source: Cancers (Basel). 2022 Oct 26;14(21):5240. doi: 10.3390/cancers14215240 (PMC9655638; doi:10.3390/cancers14215240)
Supplement: Supplementary file 1 [file cancers-14-05240-s001.zip › Figure S8.pdf]

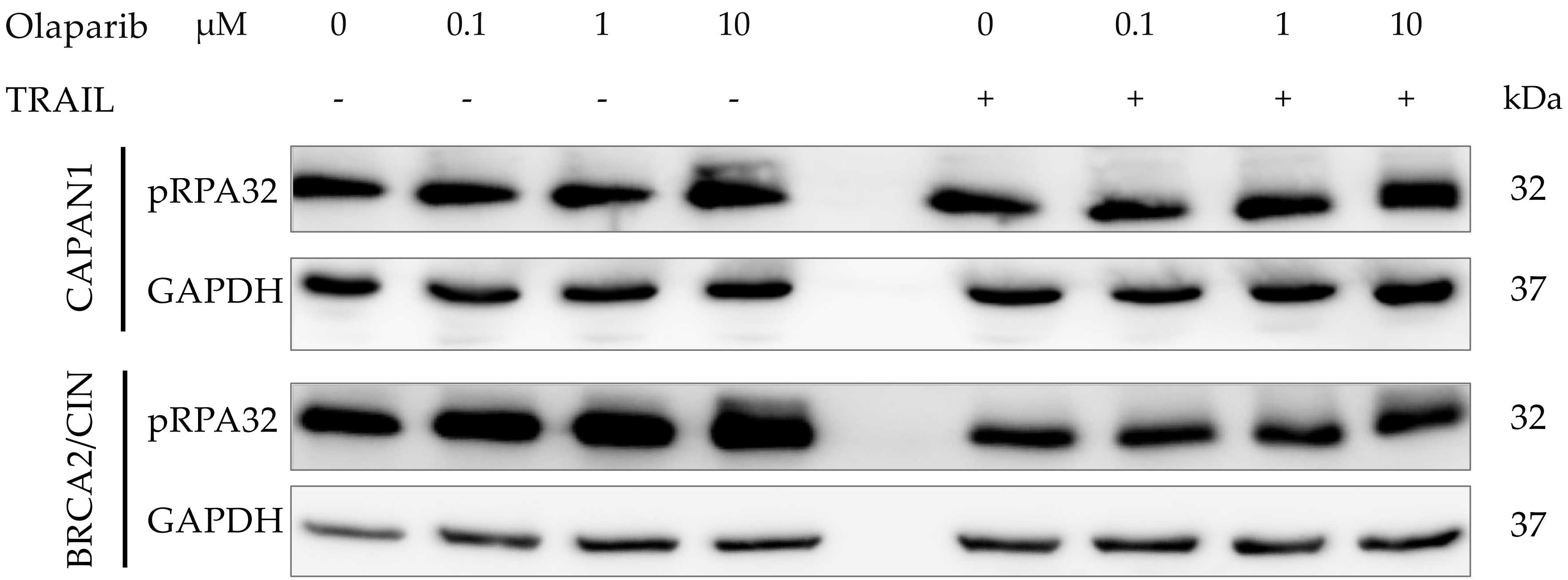

Figure S8. Western blot analysis to assess the expression levels of pRPA32 in BRCA2 deficient CAPAN1 vs. wild-type BRCA2 expressing BRCA2/CIN treated with various doses of olaparib alone or in combination with TRAIL (10 ng/ml) for 48 h. Data shows representative results from three experiments. GAPDH served as loading control.
